# A reappraisal of lymph node dissection in colorectal cancer during primary surgical resection

**DOI:** 10.1186/s12957-020-01863-2

**Published:** 2020-05-17

**Authors:** Yen-Jen Chen, Shin-Ting Yeh, Ping-Sheng Kao, Liang-Hung Ou, Chen-Sung Lin

**Affiliations:** 1 Department of Surgery, Taipei Hospital, Ministry of Health and Welfare, No. 127, Su-Yuan Road, Hsin-Chuang Dist., New Taipei City, Taiwan; 2 Division of General Surgery, Department of Surgery, Taipei Veterans General Hospital, No. 201, Sec. 2, Shi-Pai Road, Bei-Tou Dist., Taipei, Taiwan; 3 Faculty of Medicine, National Yang-Ming University, No. 155, Sec. 2, Li-Nong Street, Bei-Tou Dist., Taipei, Taiwan; 4 Department of Gerontological Health Care, and College of Nursing, National Taipei University of Nursing and Health Sciences, No. 365, Ming-Te Road, Bei-Tou Dist., Taipei, Taiwan; 5 School of Life Science, National Taiwan Normal University, No. 88, Sec. 4, Ting-Chow Road, Wen-Shan Dist., Taipei, Taiwan; 6 Center for General Education, Kainan University, No.1, Kai-Nan Road, Lu-Zhu Dist., Taoyuan City, Taiwan

**Keywords:** Colorectal cancer (CRC), lymph node dissection (LND), Total dissected lymph nodes (TDLNs), Prognosis

## Abstract

**Purpose:**

Controversy exists regarding the extent to which lymph node dissection (LND) should be performed for operable colorectal cancers (CRCs) during primary surgical resection. We reappraised the role of LND in CRCs.

**Methods:**

Seventy-three CRC patients (mean age, 65.3 years; 43 males) undergoing primary surgical resection at Taipei Hospital, Ministry of Health and Welfare, Taiwan, within a 3-year period were retrospectively analyzed. Their pathological T/N/M statuses and cancer stages were defined according to the American Joint Committee on Cancer (AJCC) 8th edition staging system. The numbers of total dissected lymph nodes (TDLNs), positive dissected lymph nodes (PDLNs), and negative dissected lymph nodes (NDLNs) for each CRC patient were recorded in detail (TDLNs = PDLNs + NDLNs). Possible prognostic variables were evaluated.

**Results:**

An advanced N status (N1/N2 vs. N0; HR, 5.749/17.677 vs. 1.000; *p* = 0.056/0.009) and M1 status (M1 vs. M0; HR, 7.517 vs. 1.000; *p* = 0.010) were independent variables for a poor prognosis. For all 73 CRC patients (*p* = 0.030), as well as T2 CRC patients (*p* = 0.061), those with > 15 TDLNs tended to have more PDLNs than those with ≤ 15 TDLNs. For 42 N(+) CRC patients (*p* = 0.007), as well as N2 CRC patients (*p* = 0.011), those with > 21 TDLNs tended to have more PDLNs than those with ≤ 21 TDLNs.

**Conclusion:**

For CRC patients undergoing primary surgical resection, the number of TDLNs influences the accuracy of nodal staging. A minimum of 15 TDLNs is necessary for positive lymph nodes to be identified in CRC patients, and 21 TDLNs is sufficient for the severity of the N(+) status to be distinguished in N(+) CRC patients.

## Introduction

The incidence and prevalence of colorectal cancer (CRC) have been increasing in recent decades in Taiwan [1]. In addition to perioperative radiotherapy, chemotherapy, or both, surgical resection plus lymph node dissection (LND) plays a key role in operable CRCs [2]. Currently, the T/N/M status and cancer stage defined in the American Joint Committee on Cancer (AJCC) manual, 8th edition, remain the cornerstones to classify and tailor optimal treatment modalities for CRC patients [3, 4].

Concerning metastasis in the regional lymph nodes (i.e., the nodal positive condition), the AJCC TNM staging system emphasizes the number of positive dissected lymph nodes (PDLNs). The N status (AJCC 8th edition staging system) has been classified as N0, N1a, N1b, N2a, and N2b (stepwise) based on the number of PDLNs (0, 1, 2–3, 4–6, and more than 7, respectively) because of their predictive power for survival [3, 4]. Although a minimal requirement of 12 total dissected lymph nodes (TDLNs) is suggested, controversy exists regarding the extent to which LND should be performed [3, 4]. Some researchers emphasize that extensive LND can achieve better local-regional control, eliminate undetectable lesions, and perhaps prolong survival; some researchers emphasize that extensive LND can achieve accurate N staging; and others believe that extensive LND may increase the risk of postoperative comorbidities without improving survival [5, 6].

The extent of LND, which is represented and quantified by the number of TDLNs, is crucial for an accurate N status determination. To distinguish nodal negative (N(−)) from nodal positive (N(+)) CRC patients and to further subgroup the severity of N(+) patients, LND with sufficient TDLNs appears to be mandatory. In the current study, we reappraised the impact of LND/TDLNs on CRC patients.

## Materials and methods

### Recruitment of CRC patients

This was a retrospective study, and the subjects were retrieved from a computerized database from a single medical institution, Taipei Hospital, Ministry of Health and Welfare, Taiwan, between Jan 2008 and Dec 2010. In this period of time, 82 patients underwent upfront surgical resections for colorectal tumors, including 80 primary CRCs over the colorectal region, 1 carcinoma in situ (Tis) over the transverse colon, and 1 gastrointestinal stromal tumor (GIST) over the rectum. Among the 80 CRC patients, 7 were excluded from the analysis for the following reasons: 2 were lost to follow-up after being discharged from the hospital, 3 died within 1 month after surgery, 1 had synchronous CRCs over the sigmoid colon and rectum, and 1 had synchronous CRCs over the transverse and sigmoid colons. Ultimately, a total of 73 constitutive patients with operable CRCs and no obvious distant organ metastasis during the preoperative assessment received surgical resection as the primary treatment modality. Postoperative adjuvant radiotherapy, chemotherapy, or both were scheduled if clinically indicated. The Institute Review Board of Taipei Hospital approved this study. All of the patients were completely followed until June 2019.

### Preoperative workup

Preoperative workup included plain chest radiography, abdominal computed tomography (CT) scans from the lower chest to pelvis, a colorectal endoscopic examination, a complete blood count and cell differentials of leukocytes in the peripheral blood, routine urine tests, blood biochemistries, and EKG/cardiac sonography to assess each patient’s general and oncological conditions. A whole-body bone scan or a CT/MRI scan of the brain was performed if clinically indicated. These patients were subjected to surgical resection if they agreed and had no contraindication for surgical resection.

### Pathological examination

After examination by a pathologist, the surgical-pathological T/N/M statuses of the 73 CRC patients were confirmed and revised according to the AJCC 8th edition staging system [3, 4]. In addition, the maximal tumor diameter, primary tumor location, conditions of lymphatic vessel invasion, venous vessel invasion or perineural invasion, and histological grade of cancer cell malignancy were recorded if available.

Concerning the N status, we recoded the numbers of TDLNs and PDLNs for all CRC patients. We defined the positive rate as the number of PDLNs divided by the number of TDLNs (= PDLNs/TDLNs, %) for each CRC patient. We also defined the number of negative dissected lymph nodes (NDLNs) as the number of TDLNs minus the number of PDLNs (NDLNs = TDLNs − PDLNs) for each CRC patient.

### Prognostic variables

The potential and reported prognostic variables, including sex, age, and maximal tumor diameter, as well as the pathological findings, were recorded and analyzed. The impacts of TDLNs and NDLNs were also evaluated.

### Statistical analysis

SPSS statistical software version 17 (SPSS Inc., Chicago, IL) was used for data analyses in this retrospective study. The continuous variables between two or among three groups were compared using Student’s *t* test/the Mann–Whitey *U* test or ANOVA/the Kruskal-Wallis *H* test when appropriate. The categorical variables between two groups were compared using the chi-square test. Overall survival was measured from the date of surgery to the date of death or the last follow-up in June 2019. Survival curves of the patients were calculated and plotted by the Kaplan–Meier method. The log-rank test was used to compare survival probabilities among different levels within each categorical variable, and the univariate Cox proportional hazards regression method was used to investigate their relative hazard ratios (HRs). In addition to sex and age, variables associated with survival probability at a significance level of 0.1 or less in the log-rank test were also included in the multivariate Cox proportional hazards regression model. The optimal cutoff number of TDLNs to detect the N2b status (i.e., 7 or more PDLNs, ≤ 7 vs. > 7) among nodal positive N(+) CRC patients was determined by receiver operating characteristic (ROC) curves through the area under the curve (AUC) and the Youden index. Significance was defined as *p* < 0.05.

## Results

### Demographic data

A total of 73 CRC patients (female/male, 30/43) with a mean age of 65.3 years were retrospectively evaluated, and their demographic data are listed in Table 1. Concerning the primary tumor locations, there were 4 (5.5%), 13 (17.8%), 15 (20.5%), 6 (8.2%), 19 (26.0%), and 16 (21.9%) in the cecum, ascending colon, transverse colon, descending colon, sigmoid colon, and rectum, respectively. Concerning the types of surgical resections, there were 4 (5.5%), 25 (34.2%), 6 (8.2%), 9 (12.3%), 22 (30.1%), 2 (2.7%), 2 (2.7%), and 3 (4.1%) CRC patients undergoing segmental resection, right hemicolectomy, left hemicolectomy, anterior resection, lower anterior resection, subtotal colectomy, abdominal-perineal resection, and Hartmann’s procedure, respectively. Concerning the pathological T/N/M status and cancer stage, there were 4 (5.5%)/9 (12.3%)/56 (76.7%)/4 (5.5%) patients with the T1/T2/T3/T4 status, respectively; 31 (42.5%)/17 (23.3%)/25 (34.2%) patients with the N0/N1/N2 status, respectively; 67 (91.8%)/6 (8.2%) patients with the M0/M1 status, respectively; and 12 (16.4%)/19 (26.0%)/36 (49.3%)/6 (8.2%) patients in stages I/II/III/IV, respectively. The mean maximal tumor diameter was 5.1 cm, and 22 (30.1%), 11 (15.1%), and 4 (5.5%) tumors had lymphatic vessel invasion, venous vessel invasion, and perineural invasion, respectively. Concerning the histological grade, there were 51 (69.9%), 16 (21.9%), and 6 (8.2%) CRCs presenting low, intermediate, and high grades of cancer cell malignancy, respectively. Concerning the distribution of TDLNs, the mean, 25th percentile, 50th percentile (median), and 75th percentile were 20.7, 10, 19, and 28.5, respectively. Concerning the distribution of PDLNs and the positive rate, their means were 3.6 and 18.1%, respectively. Concerning the distribution of NDLNs, the mean, 25th percentile, 50th percentile (median), and 75th percentile were 17.2, 9, 15, and 26.5, respectively. The mean overall survival and follow-up periods were 91.6 and 70.0 months, respectively.

**Table 1:** Demographic data of the 73 CRC patients

Variables	Mean ± SD/number (%)
Gender	
Female/Male	30 (41.1)/43 (58.9)
Age (years)	65.3 ± 11.2
Tumor location	
Cecum	4 (5.5)
Ascending colon	13 (17.8)
Transverse colon	15 (20.5)
Descending colon	6 (8.2)
Sigmoid colon	19 (26.0)
Rectum	16 (21.9)
Type of surgical resection	
Segmental resection	4 (5.5)
Right hemicolectomy	25 (34.2)
Left hemicolectomy	6 (8.2)
Anterior resection	9 (12.3)
Lower anterior resection	22 (30.1)
Sub-total colectomy	2 (2.7)
Abdominal-perineal resection	2 (2.7)
Hartmann’s procedure	3 (4.1)
Tumor configuration	
Exophytic/ulcerative	25 (34.2)/48 (65.8)
Pathological findings	
T/N/M status and cancer stage, AJCC8th	
T-status	
T1/T2/T3/T4	4 (5.5)/9 (12.3)/56 (76.7)/4 (5.5)
N-status	
N0/N1/N2	31 (42.5)/17 (23.3)/25 (34.2)
M-status	
M0/M1	67 (91.8)/6 (8.2)
Stage	
I/II/III/IV	12 (16.4)/19 (26.0)/36 (49.3)/6 (8.2)
Maximal tumor diameter (cm)	5.1 ± 2.6
Lymphatic vessel invasion	
No/yes/not analyzed	42 (57.5)/22 (30.1)/9 (12.3)
Venous vessel invasion	
No/yes/not analyzed	52 (71.2)/11 (15.1)/10 (13.7)
Perineural invasion	
No/yes/not analyzed	53 (72.6)/4 (5.5)/16 (21.9)
Histological grade	
Low/intermediate/high grade	51 (69.9)/16 (21.9)/6 (8.2)
No. of TDLNs (25, 50, and 75 percentile)	20.7 ± 11.8 (10, 19, and 28.5)
No. of PDLNs (25, 50, and 75 percentile)	3.6 ± 5.2 (0, 1 and 5)
Positive rate	18.1 ± 23.6
No. of NDLNs (25, 50, and 75 percentile)	17.2 ± 11.2 (9, 15, and 26.5)
Follow-up period (mean, 95%CI) (months)	70.0 (59.5–80.5)
Overall survival (mean, 95%CI) (months)	91.6 (78.6–105.5)

*CRC* colorectal cancer, *No*. number, *TDLNs* total dissected lymph nodes, *PDLNs* positive dissected lymph nodes, *NDLNs* negative dissected lymph nodes, *Positive rate* No. PDLNs/No. TDLNs, %, *SD* standard deviation

### Prognostic variables and their HRs for all CRC patients

Among the 73 CRC patients, we found that the T status (*p* = 0.074), N status (*p* < 0.001), M status (*p* < 0.001), stage (*p* < 0.001), lymphatic vessel invasion (*p* = 0.001), venous vessel invasion (*p* = 0.001), perineural invasion (*p* = 0.037), histological grade of cancer cell malignancy (*p* = 0.022), and numbers of NDLNs (*p* = 0.071) were prognostic variables for survival (Table 2).

**Table 2:** Prognostic variables, survivals, and their hazard ratios (HRs) of the 73 CRC patients

Prognostic variables	Survival differences		Cox’s proportional hazards regression			
	Survival, months	Log-rank	Univariate		Multivariate	
	Mean (95% CI)	*p* value	HR (95% CI)	*p* value	HR (95% CI)	*p* value
Gender		0.361				
Female (*n* = 30)	81.3 (61.3–101.2)		1.000		1.000	
Male (*n* = 43)	97.5 (81.2–113.9)		0.715 (0.347–1.475)	0.364	2.354 (0699–7.929)	0.167
Age (years)		0.592				
≤65 (*n* = 44)	87.1 (70.9–103.2)		1.003 (0.973–1.035)*	0.649	1.039 (0.981–1.102)*	0.193
>65 (*n* = 29)	94.8 (74.6–115.1)		-		-	
Maximal tumor diameter (cm)		0.132				
≤5 (*n* = 44)	94.2 (79.4–108.9)		1.000			
>5 m (*n* = 29)	78.4 (57.4–99.5)		1.726 (0.841–3.544)	0.137		
T-status		0.074				
T1/T2 (*n* = 13)	114.5 (95.8–133.2)		1.000		1.000	
T3 (*n* = 56)	87.9 (73.0–102.9)		3.221 (0.762–13.612)	0.112	0.431 (0.067–2.768)	0.375
T4 (*n* = 5)	51.4 (9.8–92.9)		7.100 (1.179–42.754)	0.032	0.316 (0.023–4.304)	0.387
N-status		< 0.001				
N0 (*n* = 31)	127.1 (115.9–138.3)		1.000		1.000	
N1 (*n* = 17)	86.0 (62.3–109.6)		5.267 (1.396–19.863)	0.014	5.749 (0.959–34.480)	0.056
N2 (*n* = 25)	50.9 (31.0–70.8)		12.896 (3.793–43.843)	< 0.001	17.677 (2.048–152.605)	0.009
M-status		< 0.001				
M0 (*n* = 67)	98.5 (85.6–111.4)		1.000		1.000	
M1 (*n* = 6)	16.2 (3.7–28.7)		7.708 (2.957–20.095)	< 0.001	7.517 (1.626–34.756)	0.010
Stage		< 0.001				
I (*n* = 12)	113.6 (93.3–133.9)		1.000			
II (*n* = 19)	132.2 (122.2–142.2)		0.284 (0.026–3.128)	0.303		
III (*n* = 36)	74.6 (56.9–92.3)		4.100 (0.959–17.521)	0.057		
IV (*n* = 6)	16.2 (3.7–28.7)		18.011 (3.476–93.338)	0.001		
Lymphatic vessel invasion		0.001				
No (*n* = 42)	100.1 (86.7–113.6)		1.000		1.000	
Yes (*n* = 22)	52.8 (31.9–73.6)		3.276 (1.509–7.114)	0.003	0.832 (0.226–3.060)	0.782
Venous vessel invasion		0.001				
No (*n* = 52)	94.4 (81.1–107.7)		1.000		1.000	
Yes (*n* = 11)	39.2 (16.8–61.6)		3.782 (1.590–8.993)	0.003	2.828 (0.398–20.120)	0.299
Perineural invasion		0.037				
No (*n* = 53)	90.5 (76.7–104.2)		1.000		1.000	
Yes (*n* = 4)	33.7 (0.0–72.0)		3.402 (0.996–11.625)	0.051	1.702 (0.192–15.084)	0.633
Histological grade		0.022				
Low grade (*n* = 51)	94.9 (81.0–108.8)		1.000		1.000	
Intermediate/high grade (*n* = 22)	71.2 (48.8–93.6)		2.266 (1.103–4.656)	0.026	2.332 (0.814–6.680)	0.115
Tumor configuration		0.104				
Exophytic (*n* = 25)	104.1 (87.9–120.3)		1.000			
Ulcerative (*n* = 48)	82.7 (66.1–99.3)		1.991 (0.853–4.646)	0.111		
No. of TDNLs		0.448				
≤ 10 (25 percentile, *n* = 20)	88.1 (66.0–110.3)		1.783 (0.548–5.803)	0.337		
10–29 (25–75 percentile, *n* = 37)	85.1 (66.4–103.7)		1.998 (0.671–5.950)	0.214		
> 29 (75 percentile, *n* = 16)	97.7 (74.8–121.1)		1.000			
No. of NDLNs		0.071				
≤ 9 (25 percentile, *n* = 22)	69.8 (48.1–91.5)		2.661 (0.956–7.405)	0.061	0.727 (0.153–3.448)	0.688
9–27 (25–75 percentile, *n* = 33)	98.3 (79.0–117.5)		1.300 (0.451–3.752)	0.627	0.406 (0.074–2.223)	0.299
> 27 (75 percentile, *n* = 18)	97.4 (76.5–118.2)		1.000		1.000	

*CRC* colorectal cancer, *No.* number, *TDLNs* total dissected lymph nodes, *NDLNs* negative dissected lymph nodes, *SD* standard deviation, *CI* confidence interval

^*^Considered as a continuous variable

The univariate Cox proportional hazards regression model revealed that patients with an advanced T status (T4, HR = 7.100, 95% CI = 1.179–42.754, *p* = 0.032; T3, HR = 3.221, 95% CI = 0.762–13.612, *p* = 0.112), advanced N status (N2, HR = 12.896, 95% CI = 3.793–43.843, *p* < 0.001; N1, HR = 5.267, 95% CI = 1.396–19.863, *p* = 0.014), M1 status (HR = 7.708, 95% CI = 2.957–20.095, *p <* 0.001), late cancer stage (stage IV, HR = 18.011, 95% CI = 3.476–93.338, *p* = 0.001; stage III, HR = 4.100, 95% CI = 0.959–17.521, *p* = 0.057; stage II, HR = 0.284, 95% CI = 0.026–3.128, *p* = 0.303), lymphatic vessel invasion (HR = 3.276, 95% CI = 1.509–7.114, *p* = 0.003), venous vessel invasion (HR = 3.782, 95% CI = 1.590–8.993, *p* = 0.003), perineural invasion (HR = 3.402, 95% CI = 0.996–11.625, *p* = 0.051), a high histological grade of cancer cell malignancy (HR = 2.266, 95% CI = 1.103–4.656), and few numbers of NDLNs (≤9, HR = 2.661, 95% CI = 0.956–7.405, *p* = 0.061; 9–27, HR = 1.300, 95% CI = 0.451–3.752, *p* = 0.627) tended to have poor survival and high HRs (Table 2).

**Fig. 1:**
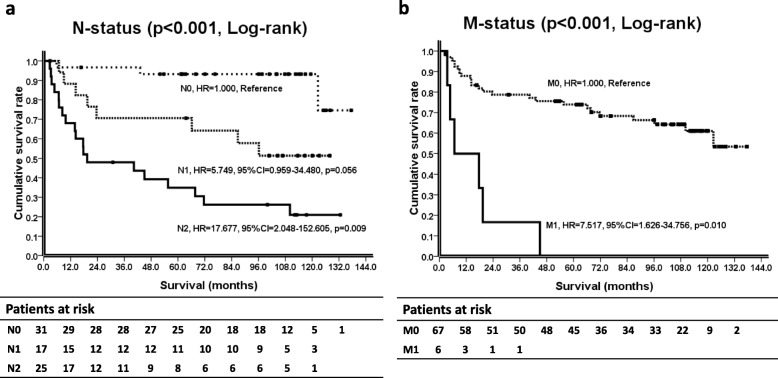
Kaplan–Meier survival curves, *p* values (log-rank test), HRs (including 95% CIs, multivariate Cox proportional hazards regression), and patients at risk based on two independent factors in CRC patients, the N status (1, **a**) and M status (1, **b**), are illustrated

We incorporated the potential variables with a log-rank *p ≤* 0.1, along with sex and age, into the multivariate Cox regression proportional hazards analysis and found that an advanced N status (N2, HR = 17.677, 95% CI = 2.048–152.605, *p* = 0.009; N1, HR = 5.749, 95% CI = 0.959–34.480, *p* = 0.056) (Fig. 1a) and M1 status (HR = 7.517, 95% CI = 1.626–34.756, *p* = 0.010) (Fig. 1b) were independently associated with a poor prognosis and high HRs in this cohort (Fig. 1, Table 2).

### Role of TDLNs/NDLNs in N(−) CRC patients

The number of TDLNs is equal to the number of NDLNs in N(−) CRC patients. For all CRC patients, we determined that few NDLNs (*p* = 0.071, Table 2) tended to be associated with poor survival, and we tested the cutoff number 15, the median number of NDLNs of 73 CRC patients (Table 1), to evaluate its influence among 31 N(−) CRC patients. Among the 31 N(−) CRC patients, 3 (9.7%) died during the follow-up period, all of whom had nonsignificantly fewer TDLNs/NDLNs than did the other 28 patients who remained alive (13.0 ± 12.3 vs. 20.7 ± 11.5, *p* = 0.181, Table 3, Part I). Two of the 3 N(−) CRC patients who died had ≤ 15 TDLNs, and 1 had > 15 TDLNs (*p* = 0.431, Table 3, Part II). In addition, N(−) CRC patients with ≤ 15 TDLNs had nonsignificantly lower 1-/2-/5-/10-/12-year survival rates and unobvious shorter survival times than those with > 15 TDLNs (92.3%, 92.3%, 92.3%, 92.3%, and 61.5% vs. 100.0%, 93.8%, 93.8%, 93.8%, and 93.8%, respectively, and 117.0 vs. 131.6 months, respectively, *p* = 0.529, Table 3, Part II). In brief, 15 TDLNs seems adequate for N(−) CRC patients, but this finding needs further validation.

**Table 3:** Differences in cumulative survival rates among the 31 N(−) CRC patients according to the current survival status and the cutoff number of TDLNs (NDLNs) of 15

Part I	Current survival status		
	Dead (*n* = 3)	Alive (*n* = 28)	*p* value
No. of TDLNs (=NDLNs) (mean ± SD)	13.0 ± 12.3	20.7 ± 11.5	0.181*
Follow-up periods (mean ± SD)(month)	57.1 ± 59.7	89.8 ± 33.9	0.316*
Cumulative survival rate (%)			
1 year	67.7%	100.0%	
2 years	67.7%	100.0%	
5 years	33.3%	100.0%	
10 years	33.3%	100.0%	
12 years	0.0%	100.0%	
Part II	TDLNs (=NDLNs)		
	≤ 15 (*n* = 14)	> 15 (*n* = 17)	*p* value
No. of deaths (*n* = 3)	2	1	0.431**
Follow-up periods (mean ± SD) (month)	87.5 ± 41.4	86.0 ± 43.2	0.912*
Survivals (mean, 95%CI) (month)	117.0 (99.3–134.7)	131.6 (120.4–142.8)	0.529***
Cumulative survival rate (%)			
1 year	92.3%	100.0%	
2 years	92.3%	93.8%	
5 years	92.3%	93.8%	
10 years	92.3%	93.8%	
12 years	61.5%	93.8%	

*CRC* colorectal cancer, *No.* number, *TDLNs* total dissected lymph nodes, *NDLNs* negative dissected lymph nodes, *SD* standard deviation, *CI* confidence interval

*Student’s *t* test/Mann–Whitey *U* test

**Chi-square test

***Log-rank

### Distributions of TDLNs, PDLNs, and NDLNs and the positive rate for all CRC patients, CRC patients with ≤ 15 TDLNs, and CRC patients with > 15 TDLNs according to the pathological T status

The mean numbers of PDLNs among all 73 CRC patients with the T1, T2, T3, and T4 status were 0.0, 0.1, 4.1, and 7.0, respectively (*p* = 0.001), and the positive rates were 0.0%, 0.3%, 21.8%, and 24.1%, respectively (*p* = 0.001) (Table 4, Part I). The numbers of TDLNs (*p* = 0.116) and NDLNs (*p* = 0.359) among CRC patients with the T1, T2, T3, and T4 statuses were not obviously different, suggesting that these LNDs were performed indiscriminately (Table 4, Part I).

**Table 4:** Distributions of TDLNs, PDLNs, NDLNs, and positive rate for overall CRC patients, CRC patients with ≤ 15 TDLNs, and CRC patients with > 15 TDLNs according to the pathological T status among the 73 CRC patients

Part I	Overall (*n* = 73)	T1 (*n* = 4)	T2 (*n* = 9)	T3 (*n* = 56)	T4 (*n* = 4)	*p* value*
No. of TDLNs (Mean ± SD)	20.7 ± 11.8	21.5 ± 11.3	13.3 ± 10.5	21.3 ± 11.6	29.5 ± 12.9	0.116
No. of PDLNs (Mean ± SD)	3.6 ± 5.2	0.0 ± 0.0	0.1 ± 0.3	4.1 ± 5.4	7.0 ± 6.4	0.001
Positive rate (Mean ± SD, %)	18.1 ± 23.6	0.0 ± 0.0	0.3 ± 1.0	21.8 ± 24.6	24.1 ± 23.1	0.001
No. NDLNs (Mean ± SD)	17.2 ± 11.2	21.5 ± 11.3	13.2 ± 10.3	17.1 ± 11.3	22.5 ± 13.5	0.359
Part II	No. of PDLNs (Mean ± SD)					
	Overall (*n* = 73)	T1 (*n* = 4)	T2 (*n* = 9)	T3 (*n* = 56)	T4 (*n* = 4)	
No. of TDLNs ≤ 15 (*n* = 28, T1/T2/T3/T4, 2/7/19/0)	1.9 ± 3.0	0.0 ± 0.0	0.0 ± 0.0	2.8 ± 3.3	-	
No. of TDLNs > 15 (*n* = 45, T1/T2/T3/T4, 2/2/37/4)	4.6 ± 5.9	0.0 ± 0.0	0.5 ± 0.7	4.8 ± 6.1	7.0 ± 6.4	
*p* value**	0.030	1.000	0.061	0.190	-	
Part III	Positive rate (mean ± SD, %)					
	Overall (*n* = 73)	T1 (*n* = 4)	T2 (*n* = 9)	T3 (*n* = 56)	T4 (*n* = 5)	
No. of TDLNs ≤ 15 (*n* = 28, T1/T2/T3/T4, 2/7/19/0)	19.5 ± 27.5	0.0 ± 0.0	0.0 ± 0.0	28.8 ± 29.3	-	
No. of TDLNs > 15 (*n* = 45, T1/T2/T3/T4, 2/2/37/4)	17.2 ± 21.0	0.0 ± 0.0	1.5 ± 2.1	18.2 ± 21.4	24.1 ± 23.1	
*p* value**	0.686	1.000	0.061	0.131	-	
Part IV	No. of N DLNs (Mean ± SD)					
	Overall (*n* = 73)	T1 (*n* = 4)	T2 (*n* = 9)	T3 (*n* = 56)	T4 (*n* = 5)	
No. of TDLNs ≤ 15 (*n* = 31, T1/T2/T3/T4, 2/7/19/0)	7.7 ± 4.2	12.0 ± 4.2	8.4 ± 4.1	7.0 ± 4.1	-	
No. of TDLNs > 15 (*n* = 45, T1/T2/T3/T4, 2/2/37/4)	23.1 ± 10.2	31.0 ± 1.4	30.0 ± 4.2	22.4 ± 10.2	22.5 ± 13.5	
*p* value**	< 0.001	0.027	0.040	< 0.001	-	

*No.* number, *TDLNs* total dissected lymph nodes, *PDLNs* positive dissected lymph nodes, *NDLNs* negative dissected lymph nodes, *Positive rate* No. PDLNs/No. TDLNs, %, *SD* = Standard deviation

*Compared among T1, T2, T3, and T4 status, ANOVA/Kruskal-Wallis *H* test

**Compared between TDLN≤ 15 and TDLN> 15, Student’s *t* test/Mann–Whitey *U* test

Concerning the number of PDLNs, those with > 15 TDLNs tended to have more PDLNs than those with ≤ 15 TDLNs (*p* = 0.030, 4.6 ± 5.9 vs. 1.9 ± 3.0), especially those with the T2 status (*p* = 0.061, 0.5 ± 0.7 vs. 0.0 ± 0.0) (Table 4, Part II). This result denotes that a minimum of 15 TDLNs is highly required to detect the N(+) status in CRC patients. Such a difference was not observed when analyzing the distribution of the positive rate (*p* = 0.686, Table 4, Part III).

Concerning the number of NDLNs, those with > 15 TDLNs tended to have more NDLNs than those with ≤ 15 TDLNs (*p <* 0.001, 23.1 ± 10.2 vs. 7.7 ± 4.2), regardless of the T1 (*p* = 0.027, 31.0 ± 1.4 vs. 12.0 ± 4.2), T2 (*p* = 0.040, 30.0 ± 4.2 vs. 8.4 ± 4.1), or T3 (*p <* 0.001, 22.4 ± 10.2 vs. 7.0 ± 4.1) status (Table 4, Part IV).

### Distributions of the mean numbers of TDLNs, PDLNs, and NDLNs and the mean positive rates in 42 N(+) CRC patients based on the number of TDLNs

The N2b status, the most advanced N status defined in the current AJCC 8th edition staging system, is defined as 7 or more PDLNs. We tested various cutoff points for TDLNs on ROC curves (AUC = 0.723, 95% CI = 0.560–0.886, *p* = 0.022), and 20.5 (i.e., 21, after rounding up 20.5) resulted in the highest Youden index of 0.459 (sensitivity = 0.769, specificity = 0.690) to distinguish between ≤ 7 and > 7 PDLNs for all 42 N(+) CRC patients. As shown in Table 5, the positive rate dropped sharply from 28.3 to 24.5% when ≥ 23/24 TDLNs was used for the 42 N(+) CRC patients. This result denotes the limitation of the positive rate during each LND, and the peak number of TDLNs did not exceed 24. Furthermore, 19 of 42 N(+) CRC patients had ≥ 21 TDLNs, and the peak positive rate (29.8%) was higher in these patients than in those with ≥ 10 ~ ≥30 TDLNs. Therefore, we propose that 21 TDLNs is sufficient.

**Table 5:** Distributions of mean numbers of TDLNs, PDLNs, and NDLNs and the mean positive rates in 42 N(+) CRC patients based on the number of TDLNs

Sub-groups	No. of accumulative cases	No. of TDLNs (mean)	No. of PDLNs (mean )	Positive rate (Mean, %)	No. of NDLNs (Mean)
Overall	42				
TDLNs ≥ 4	42	21.2	6.2	31.5	15.0
TDLNs ≥ 6	41	21.6	6.3	31.0	15.4
TDLNs ≥ 7	40	22.0	6.4	31.4	15.6
TDLNs ≥ 9	38	22.8	6.7	32.3	16.1
TDLNs ≥ 10	34	24.4	6.9	29.5	17.6
TDLNs ≥ 13	31	25.8	7.2	29.1	18.6
TDLNs ≥ 14	30	26.3	7.4	29.9	18.8
TDLNs ≥ 16	28	27.1	7.4	27.7	19.8
TDLNs ≥ 17	26	28.0	7.7	28.6	20.3
TDLNs ≥ 18	24	28.9	7.8	27.8	21.1
TDLNs ≥ 19	23	29.4	8.1	28.5	21.3
TDLNs ≥ 20	20	31.0	8.6	28.8	22.4
TDLNs ≥ 21*	19	31.5	8.9	29.8	22.6
TDLNs ≥ 23	17	32.8	8.9	28.3	23.9
TDLNs ≥ 24	15	34.1	8.3	24.5	25.7
TDLNs ≥ 25	14	34.8	8.2	23.3	26.6
TDLNs ≥ 27	12	36.4	8.4	22.5	28.0
TDLNs ≥ 28	10	38.3	9.4	24.4	28.9
TDLNs ≥ 29	9	39.4	8.7	20.8	30.8
TDLNs ≥ 30	8	40.8	9.5	22.5	31.3
TDLNs ≥ 34	7	42.3	10.7	25.3	31.6
TDLNs ≥ 35	6	43.7	12.3	29.0	31.3
TDLNs ≥ 36	5	45.4	12.2	27.3	33.2
TDLNs ≥ 41	4	47.8	10.5	21.0	37.3
TDLNs ≥ 47	3	50.0	13.0	25.5	37.0
TDLNs ≥ 51	2	51.5	17.0	33.0	34.5
TDLNs ≥ 52	1	52.0	22.0	42.3	30.0

*TDLNs* total dissected lymph nodes, PDLNs positive dissected lymph nodes, *NDLNs* negative dissected lymph nodes, *Positive rate* No. PDLNs/No. TDLNs, %, *SD* standard deviation, *CI* confidence interval, *ROC* receiver operating characteristic, *AUC* area under the curve

*We tested various cutoff points for TDLNs on ROC curves (AUC = 0.723, 95%CI = 0.560–0.886, *p* = 0.022) and 20.5 (i.e., 21, after rounding up 20.5) had the highest Youden index of 0.459 (sensitivity = 0.769, specificity = 0.690) to distinguish PDLNs ≤ 7 or PDLNs > 7

### Distributions of TDLNs, PDLNs, and NDLNs and the positive rate for N(+) CRC patients, N(+) CRC patients with ≤ 21 TDLNs, or N(+) CRC patients with > 21 TDLNs according to the pathological N status

For the 42 N(+) CRC patients, the distributions of the number of TDLNs (*p* = 0.199) and NDLNs (*p* = 0.409) were not different between those with the N1 (*n* = 17) status and those with the N2 (*n* = 25) status, suggesting that these LNDs were performed indiscriminately. The N2 CRC patients had more PDLNs (*p <* 0.001, 9.3 ± 5.2 vs. 1.6 ± 0.7) and higher positive rates (*p <* 0.001, 44.1 ± 20.5% vs. 12.8 ± 11.9%) than the N1 CRC patients (Table 6, Part I).

**Table 6:** Distributions of TDLNs, PDLNs, and NDLNs and the positive rate for N(+) CRC patients, N(+) CRC patients with ≤ 21 TDLNs, or N(+) CRC patients with > 21 TDLNs according to the pathological N status

Part I	Nodal positive (*n* = 42)	N1 (*n* = 17)	N2 (*n* = 25)	*p* value*
No. of TDLNs (mean ± SD)	21.2 ± 12.0	18.3 ± 10.9	23.2 ± 12.6	0.199
No. of PDLNs (mean ± SD)	6.2 ± 5.5	1.6 ± 0.7	9.3 ± 5.2	< 0.001
Positive rate (mean ± SD, %)	31.5 ± 23.3	12.8 ± 11.9	44.1 ± 20.5	< 0.001
No. NDLNs (mean ± SD)	15.0 ± 10.6	16.7 ± 10.8	13.9 ± 10.5	0.409
Part II	No. of PDLNs (mean ± SD)			
	Nodal positive (*n* = 42)	N1 (*n* = 17)	N2 (*n* = 25)	
No. of TDLNs ≤ 21 (*n*=25, N1/N2, 12/13)	3.4 ± 3.8	1.6 ± 0.7	6.9 ± 3.6	
No. of TDLNs > 21 (*n*=17, N1/N2, 5/12)	8.9 ± 6.6	1.6 ± 0.9	11.9 ± 5.4	
*p* value**	0.007	0.907	0.011	

*TDLNs* total dissected lymph nodes, *PDLNs* positive dissected lymph nodes, *NDLNs* negative dissected lymph nodes, *Positive rate* No. PDLNs/No. TDLNs, %, *SD* standard deviation

*Compared between N1 and N2 status, Student’s *t* test/Mann–Whitey *U* test

**Compared between TDLN≤21 and TDLN>21, Student’s t test/Mann–Whitey *U* test

Concerning the number of PDLNs, those with > 21 TDLNs tended to have more PDLNs than those with ≤ 21 TDLNs (*p* = 0.007, 8.9 ± 6.6 vs. 3.4 ± 3.8), especially those with the N2 (*p* = 0.011, 11.9 ± 5.4 vs. 6.9 ± 3.6) status (Table 6, Part II). Consistent with the results described above, 21 TDLNs seemed sufficient and adequate to detect the most advanced N status (N2/N2b).

## Discussion

Compatible with the reported literature, we demonstrated that an advanced N status and M1 status were independent variables related to a poor prognosis in this cohort (Fig. 1, Table 2) [1, 7]. How to set an accurate N status to predict the survival of CRC patients has become an important task. In the current study, we reappraised and focused on the impacts of TDLNs on N status staging.

Similar to gastric cancer [8] and esophageal cancer [9], the current AJCC 8th edition staging system for CRC emphasizes the number of PDLNs and a minimal requirement of 12 TDLNs for accurate N staging. According to the equation (PDLNs = TDLNs − NDLNs), PDLNs are related to the dynamic changes in TDLNs and NDLNs. Undoubtedly, we have a higher probability of detecting the N(+) status or confirming a true N(−) status if we perform extensive LND to harvest a sufficient number of TDLNs or NDLNs. Through indiscriminant TDLNs/NDLNs among different T statuses (T1 vs. T2 vs. T3 vs. T4, *p* = 0.116/0.359, Part I, Table 4), we found progressive increases in the numbers of PDLNs (*p* = 0.001) and positive rates (*p* = 0.001) in CRCs (from T1, T2, to T3, and further T4). This finding denotes a higher probability that the N(+) status could be identified in T3/T4 than in T1/T2 CRCs. In other words, the N(+) status would be underestimated as the N(−) status in T1/T2 CRCs if we do not harvest sufficient numbers of TDLNs/NDLNs for analysis.

As described in Tables 3 and 4, we highly recommended a minimal requirement of 15 TDLNs for CRC patients undergoing primary resection. However, the cutoff value of 15 differs from the suggestion in the AJCC 8th edition staging system. Different cutoff values of TDLNs were also reported in other series, and potential differences in race, surgeons, types of surgical resection, and statistics might account for such differences [10–12]. Nevertheless, the main aims of these reported articles were similar, and all paid attention to the minimal requirement of TDLNs to detect the N(+) status or to avoid understaging N(−) patients.

Another important issue is to what extent LND can be performed. As described in Tables 5 and 6, we found that 21 TDLNs seemed sufficient to detect the most advanced N(+) status, N2b, in N(+) CRC patients. Baxter et al. used the “ceiling effect” to explain the phenomenon underlying the positive rate reported in Table 5 [12, 13]. In the literature, few studies have discussed the extent of TDLNs for N(+) patients. Despite the poor prognosis associated with an advanced N2b status, chemotherapy, radiotherapy, immunotherapy, or all treatments can be tailored and advocated if the N(+) status is classified accurately.

In the literature, large numbers of NDLNs were reported to be associated with prolonged survival in patients with ESCC, gastric cancer, or CRC [14–17]. Compatible with these results, our preliminary results also demonstrated that a large number of NDLNs was related to prolonged survival among the 73 CRC patients (*p* = 0.071, subgrouped, ≤ 9, 9–25, and > 25, Table 2). This result indicates that a large number of NDLNs is related to prolonged survival in CRC patients, and a minimal requirement of 9 NDLNs has been reported by Quan et al [16]. However, the prognostic impact of NDLNs was excluded when PDLNs were defined according to the N status (AJCC 8th edition staging system, N0, N1, and N2) after multivariate Cox regression proportional hazards analysis (Table 2). Such a situation is usually encountered in clinical practice when we are concerned about nodal condition from the viewpoint of PDLNs/TDLNs simultaneously. For example, when CRC patients experience nodal conditions such as those in case A (1/10 [PDLNs/TDLNs] [N1a]) vs. case B (4/20 [N2a]), most surgeons believe that case B might have a worse prognosis due to a later N status although case B has a larger number of NDLNs. As we demonstrated, the number of PDLNs has a greater effect on survival than the number of NDLNs. In contrast, when CRC patients experience nodal conditions such as those in case C: 1/10 (N1a) vs. case D: 1/20 (N1a), most surgeons believe that the N1a status of case D is more accurate than that of case C because case C might be understaged due to fewer NDLNs. As a result, we believe that in a situation with the same number of PDLNs, a large number of NDLNs denotes more accurate N status staging. However, the minimal requirement of NDLNs remains unclear. To simplify this problem, we found that the number of NDLNs was highly related to the number of TDLNs in our cohort (*p <* 0.001, Pearson’s correlation coefficient = 0.900, data not shown). Therefore, we suggest that a minimum of 15 TDLNs is necessary to differentiate the N(−) or N(+) status in CRC patients and that 21 TDLNs seem sufficient to detect the severity of N status staging in N(+) CRC patients.

Some authors have reported that right hemicolectomy may harvest more TDLNs than other types of resection and argued that the minimal requirement of TDLNs be based on the regions of vascular pedicles to be ligated [18, 19]. Compatible with these findings, we found that the mean numbers of TDLNs harvested from segmental resection, right hemicolectomy, left hemicolectomy, anterior resection, lower anterior resection, subtotal resection, abdominal-perineal resection, and Hartmann’s procedure were 11.5, 29.0, 16.0, 22.2, 16.6, 21.0, 6.5, and 9.0, respectively, with right hemicolectomy (29.0) harvesting the largest number of TDLNs (*p <* 0.001, data not shown). Nevertheless, the mean numbers of PDLNs were 4.5, 3.2, 5.8, 1.2, 3.7, 7.0, 2.0, and 5.3, respectively, without obvious differences (*p* = 0.715, data not shown), suggesting that the type of surgery has no significant impact on N status staging. Whether the minimal requirement of TDLNs should be revised according to different vascular pedicles remains under debate. We need additional clinical data for validation in the future.

This study does offer relevant information for colorectal surgeons and oncologists. Nevertheless, there are several limitations, including the small sample size and the fact that this was a retrospective analysis from a single medical institution. More case numbers recruited from multiple centers or nationwide databases are necessary for further study.

## Conclusion

In conclusion, a sufficient number of TDLNs is an important checkpoint for the adequate N status staging of CRC patients.

## Data Availability

The datasets used and analyzed during the current study are available from the corresponding author on reasonable request.
